# Resins for Frontal Photopolymerization: Combining Depth-Cure and Tunable Mechanical Properties

**DOI:** 10.3390/ma14040743

**Published:** 2021-02-05

**Authors:** Catharina Ebner, Julia Mitterer, Joamin Gonzalez-Gutierrez, Gisbert Rieß, Wolfgang Kern

**Affiliations:** 1 Department of Polymer Engineering and Science, Chair in Chemistry of Polymeric Materials, University of Leoben, 8700 Leoben, Austria; catharina.ebner@unileoben.ac.at (C.E.); julia.mitterer@unileoben.ac.at (J.M.); wolfgang.kern@unileoben.ac.at (W.K.); 2 Department of Polymer Engineering and Science, Chair of Polymer Processing, University of Leoben, 8700 Leoben, Austria; joamin.gonzalez-gutierrez@unileoben.ac.at

**Keywords:** frontal photopolymerization, photofrontal polymerization, photobleaching, (meth) acrylate polymers, TPO-L, polyol

## Abstract

Photopolymerization has undergone significant development in recent years. It enables fast and easy processing of materials with customized properties and allows precise printing of complex surface geometries. Nevertheless, photopolymerization is mainly applied to cure thin films since the low curing depth limits the fast production of large volumes. Frontal photopolymerization (FPP) is suitable to overcome these limitations so that curing of centimeter-thick (meth)acrylic layers can be accomplished within minutes by light induction only. Prerequisites, however, are the low optical density of the resin and bleaching ability of the photoinitiator. To date, tailored FPP-resins are not commercially available. This study discusses the potential of long-chain polyether dimethacrylates, offering high-temperature resistance and low optical density, as crosslinkers in photobleaching resins and investigates the mechanical properties of photofrontally-cured copolymers. Characteristics ranging from ductile to hard and brittle are observed in tensile tests, demonstrating that deep curing and versatile material properties are achieved with FPP. Analyzed components display uniform polymerization over a depth of four centimeters in Fourier transform infrared spectroscopy and swelling tests.

## 1. Introduction

**Figure 1:**
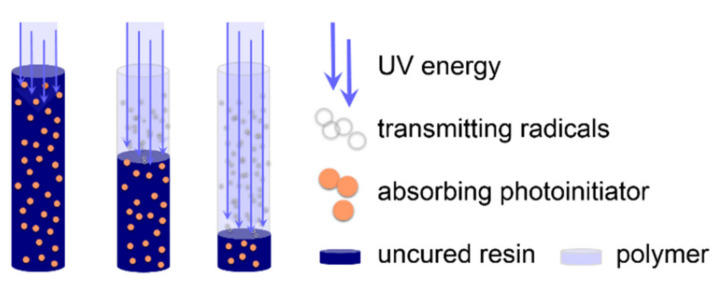
Schematic representation of a frontal photopolymerization reaction [23]. Penetration depth of light increases with radical formation, i.e., bleaching of the photoinitiator. Accordingly, a polymerization front propagates through the layer.

Light-induced polymerization offers substantial advantages in terms of time, resources, and energy efficiency. The versatile applications of photopolymerized materials include films, printing inks, dental composite resins, coatings, and 3D printed parts in additive manufacturing [1,2]. Contrary to the earlier reputation of low mechanical properties (high brittleness), high-performance photopolymers with exceptional characteristics are now produced [3,4,5,6]. In addition, advanced 3D printing technologies, such as stereolithography (SLA) or hot lithography and two-photon polymerization, realize highly complex geometries and ultra-smooth surfaces layer by layer [7,8]. Nevertheless, the maximum curing depth in photopolymerization is limited by the attenuation of light according to Beer–Lambert’s law. Therefore, applications are confined to small volumes and low curing depth in the micro to millimeter range [9]. Frontal polymerization techniques represent a powerful tool to overcome these limitations [10,11,12,13]. While in thermal frontal polymerization additional (thermal) initiators are used to cure shadow areas, frontal photopolymerization (also photofrontal polymerization) aims at curing several centimeter-thick layers by light induction only [14,15]. FPP relies on the bleaching ability of photoinitiators (PIs) whose decay products (radicals) do not hinder the absorbance of the starting molecule [16]. Consequently, light penetrates deeper into a resin, resulting in an increased depth of cure (Figure 1). In contrast to thermal frontal polymerization, FPP does not require additional initiators, making curing and reaction rates easier to control. Unless autoacceleration occurs, shadow areas remain uncured. Typically, acyl phosphine oxides, such as the trimethylbenzoyl phosphine oxide (TPO) family or 1,2-diones, including camphorquinone, serve as photobleaching initiators [17,18,19,20]. Currently, FPP is mainly used in dental restorative medicine, where only a few millimeters of a highly filled methacrylic resin are cured employing visible light-emitting diodes [21,22]. In addition, unfilled resins with low optical density have been polymerized to a maximum of 13 cm in depth [9].

Though early attempts seemed impractical and time-consuming, a recent study proves that low optical density (meth) acrylate resins polymerize up to five centimeters within minutes [23]. Prerequisites for homogeneous curing are sufficiently high intensity and energy dose of the actinic source and an appropriately selected PI concentration [24,25]. Further, the heat emanating from a strong actinic source and exothermic polymerization affects the curing process and, consequently, the properties of the resulting polymeric material [26,27,28,29,30]. In dimethacrylate-based digital light processing (DLP) 3D printing resins with high viscosity, increased printing temperature positively influenced double bond conversion, tensile strength, and modulus while requiring a shorter exposure [31]. With respect to FPP, the reduction of critical energy resulting from the increased temperature could favor deep curing by compensating for the lower exposure in deeper layers.

Although numerous practical and theoretical studies provide an ideal background for optimization, tailor-made resins explicitly designed for FPP are not yet commercially available. In this study, we mechanically characterize polymers produced from resins containing thermostable long-chain polytetrahydrofuran dimethacrylates. Properties such as Young’s modulus, tensile strength, and elongation at break are tuned by copolymerization with isobornyl methacrylate (IBOMA). In addition, Fourier transform infrared (FT-IR) spectroscopy and the determination of the gel content analyze whether components with a thickness of several centimeters polymerize homogeneously.

## 2. Materials and Methods

### 2.1. Materials

Polytetrahydrofurans of 4000 g/mol (PTHF_4000_) and 2900 g/mol (PTHF_2900_) average molecular weight were obtained from Mitsubishi Chemical Corporation (Tokyo, Japan) and Sigma Aldrich (Vienna, Austria), respectively. Methacrylic anhydride (MAA) and 2,6-di-tert-butyl-cresol (BHT) were purchased from Sigma Aldrich (Vienna, Austria) and 2,4,6-trimethylbenzoylphenyl phosphinate (TPO-L) was provided by IGM Resins (Walwijk, The Netherlands). IBOMA was purchased from TCI Germany GmbH (Eschborn, Germany). All reagents were used without further purification.

### 2.2. Polyol Modification

Modification of PTHF_2900_ and PTHF_4000_ was performed via solvent- and catalyst-free microwave-assisted transesterification as described elsewhere [23]. Both reactions were performed with one fold molar excess of MAA. The conversion was followed by FT-IR (Vertex 70, Bruker, Ettlingen, Germany) and nuclear magnetic resonance (NMR) spectroscopy (Avance III 300 MHz, Bruker, Ettlingen, Germany) [21]. In addition to α, ω-poly(tetrahydrofuran) dimethacrylate (PTHF-DM), methacrylic acid (MA) was formed as a by-product. Consequently, the bulk mixtures contained solely photopolymerizable monomers after the reaction. Both, FTIR and NMR spectra indicated full conversion of hydroxyl groups. Given the molar quantity of reacted polyol, the amount of residual MAA and MA, formed in the reaction, were calculated as percentage by mass (wt.%) contained in the mixture. PTHF_4000_-DM was used without further purification, including 7.0 wt.% MAA and 4.0 wt.% MA as copolymers. In contrast, vacuum distillation removed the excess reactant and by-product from PTHF_2900_-DM to obtain pure crosslinker.

### 2.3. Composition and Preparation of FPP-Resins

**Figure 2:**
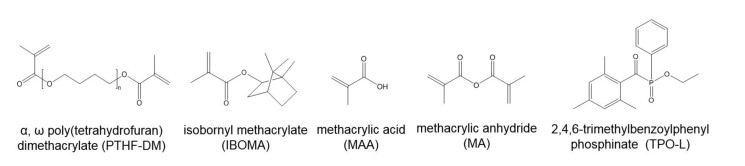
Chemical structures of methacrylate monomers and photobleaching initiator used in FPP-resins.

Figure 2 displays the chemical structures of monomers and initiator. Apart from the modified polyols, FPP-resins contained varying amounts of IBOMA and 1.0 wt.% TPO-L each (Table 1).

Prior to curing resins were exposed to ultrasonication (water bath, 15 min, 50 °C) and mixed to homogeneity via VM-200 (StateMix, Winnipeg, MB, Canada).

### 2.4. Specimen Production-Curing

For tensile tests dog-bone-shaped specimens according to ISO527-2., geometry 1BA were prepared [32]. Specimens of hexagonal shape (width 4.8 cm, depth 4 cm) were produced for FTIR analysis and swelling tests. Curing was performed in silicone molds under irradiation of a medium pressure mercury lamp (emission range between 200 and 600 nm, with the main output lines at 366, 405 and 436 nm) using a Light Hammer 6 curing system (Fusion UV Systems, Gaithersburg, MD, USA) equipped with a semi-elliptical reflector and LC6B benchtop conveyor as described elsewhere [23]. The applied intensities and the exposure dose in the sample plane were determined using UV Power Puck^®^ II radiometer (EIT Instrument Markets LLC, Leesburg, VA, USA) and are given in Table 2. The distance between the sample surface and light source was 13 cm.

Given that a single run requires only about 15 s, the entire irradiation takes no more than a few minutes. Specimens were stored overnight at 40 °C and post-cured the next day (100% power, three runs).

### 2.5. Characterization

#### 2.5.1. UV–VIS Spectroscopy

UV–VIS spectra of monomers and photoinitiator were recorded using a Cary 50 UV–VIS spectrophotometer (Varian, Waldbronn, Germany). Determination of extinction (*E*) followed the Beer–Lambert law:

(1) $$E=log_{10}\left(\frac{T_{0}}{T}\right)$$

where *T*_0_ denotes the incident transmittance, and *T* is the measured transmittance. The molar absorptivity (ε) is determined according to:

(2) $$\varepsilon \;\left(L\;mol^{-1}cm^{-1}\right)=\frac{E}{c\times d}$$

where *c* is the molar concentration and *d* is the path length.

#### 2.5.2. FTIR Spectroscopy

FTIR spectroscopy was performed with an ATR (attenuated total reflection) accessory on a Vertex 70 (Bruker, Ettlingen, Germany) spectrometer in transmission mode with an accumulation of 16 scans. Six spectra each were recorded and superimposed at depths of 1–13 mm (top), 14–26 mm (middle), and 27–40 mm (bottom).

#### 2.5.3. Swelling and Determination of Gel-Content

**Figure 3:**
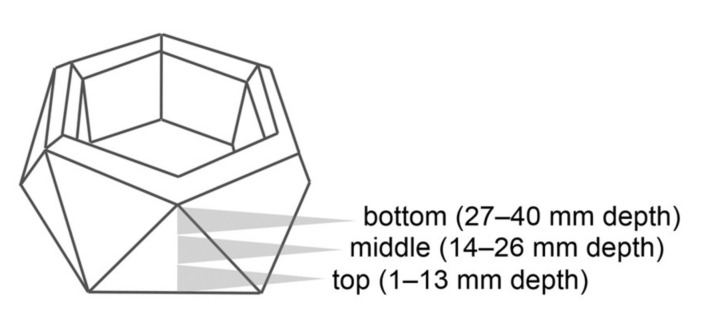
Sectioning of test specimens for swelling experiments.

Specimens produced from silicone molds with a hexagonal shape were sliced into three equally sized pieces and analyzed separately to assess the uniformity of polymerization (Figure 3). In order to detect the mass loss of hydrophilic and hydrophobic components, ethanol and toluene served as solvents for swelling experiments. Specimen parts were allowed to swell for 240 h at 40 °C.

The mass related solvent-uptake (swelling ratio *Q*) was determined according to:

(3) $$Q=\frac{w_{s}-w_{i}}{w_{i}}$$

with *w*_i_ as the initial mass of the polymeric network and *w*_s_ the mass of the swollen network. From the mass remaining after vacuum drying at 110 °C, the gel-content was determined according to:

(4) $$Gel-content\;\left(\%\right)=\;\frac{w_{d}}{w_{i}}\times 100$$

with *w*_d_ as the mass of the remaining dry network.

#### 2.5.4. Tensile Testing

Tensile tests were performed on a Zwick Z001 (Zwick/Roell, Ulm, Germany) according to ISO527-1:2019 with a test speed of 50 mm min^−1^ and 1 kN load cell [33]. The Young’s modulus was determined in the linear range between 0.05% and 0.25% elongation and measured at a test speed of 1 mm min^−1^. At least five test specimens of each formulation were tested.

## 3. Results and Discussion

### 3.1. UV–VIS Spectroscopy of Monomers and Initiator Contained in FPP-Resins

**Figure 4:**
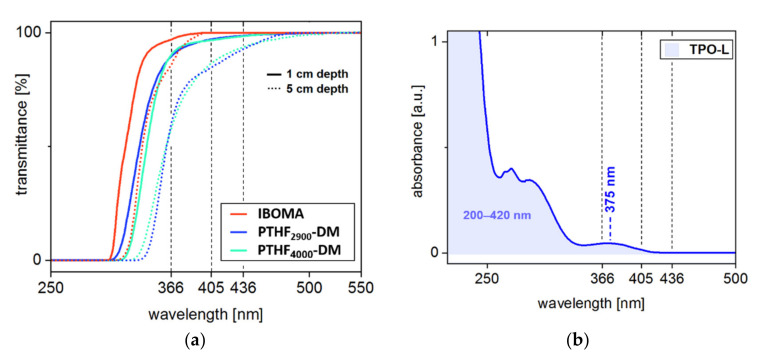
(**a**) Pure monomers are highly transmissive to the major output lines of the medium pressure mercury lamp at 366, 405, and 436 nm, respectively. With increasing film thickness, the intensity of the i-line (366 nm) is attenuated by PTHF-DM monomers to about 50% in five centimeters depth. (**b**) The absorbance maximum of TPO-L around 375 nm captures the i-line (366 nm) and the h-line (405 nm) emitted by the medium pressure mercury lamp.

In frontal photopolymerization, the initiation is driven solely by light. Thus, the amount of radicals formed at a certain depth is determined by the light energy absorbed. Assuming complete bleaching of the initiator, the attenuation of light is dominated by the molar absorptivity of the monomers and the sample depth according to Beer–Lambert’s Law. To ensure that sufficient radicals are formed, even in layers several centimeters thick, the monomers contained in the resin should not hamper the absorbance of the photoinitiator. As they are relevant for depth curing, this is particularly important for wavelengths in the long or medium wave UV (UVA, UVB) or VIS region. At one centimeter layer thickness the main output lines of the medium-pressure mercury lamp used in this study (366, 405, and 436 nm) pass through the pure monomers almost unimpeded (Figure 4a). Among the monomers used, IBOMA features the most favorable transmittance spectrum. Even in five centimeters depth, the intensity of the incident light is only slightly attenuated, with transmission at 366 nm reaching 80%. Contrarily, the increased molar absorptivity of the crosslinkers PTHF_2900_-DM and PTHF_4000_-DM could adversely affect in-depth radical formation (50% transmittance at 366 nm) by interfering with the absorption of the photoinitiator, TPO-L, around its maximum at 375 nm (Figure 4b).

Apart from the monomer structure, phenolic stabilizers such as butylated hydroxytoluene (BHT), topanol A and hydroquinone monomethyl ether (MeHQ), influence the absorption behavior of a monomer mixture. These additives tend to discolor under intense heat (e.g., forming quinones), which may also impair the absorption of the initiator [34,35].

### 3.2. Analysis of Photofrontally Cured Specimens: Through-Cure and Homogeneity

**Figure 5:**
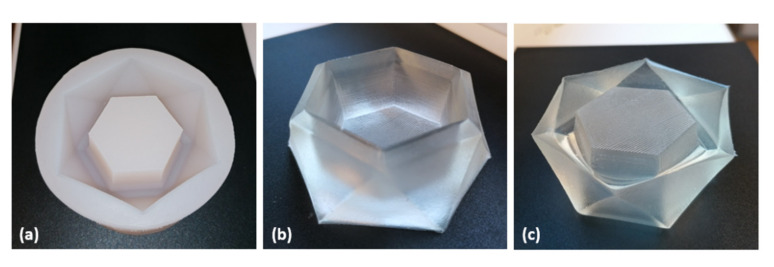
(**a**) Silicone rubber mold with four centimeters depth. (**b**) Photofrontally-cured specimen of resin R-4000_50 with a total weight of 60 g. (**c**) The grid-like structure of the hexagonal surface is accurately reproduced. The surface exposed to air appeared slightly sticky due to oxygen inhibition, whereas all other areas appeared dry and smooth.

A central objective in frontal photopolymerization is to initiate a polymerization front that uniformly cures a sample over a given depth. In contrast to thin layers, such as those produced in 3-D printing, double bond conversion of large volumes cannot be monitored in real-time via FTIR or photo-DSC. Instead, sections of the test specimen are analyzed and compared after polymerization. Using FPP-resin R-4000_50, components of hexagonal shape were cured in a silicone mold (Figure 5a). The mass of the manufactured component reached 60 g with a depth of 4.0 cm and a maximum width of 4.8 cm, whereby the grid-like inner structure was accurately reproduced (Figure 5b,c). Examples of other photofrontally-cured components are given in Figures S1 and S2.

**Figure 6:**
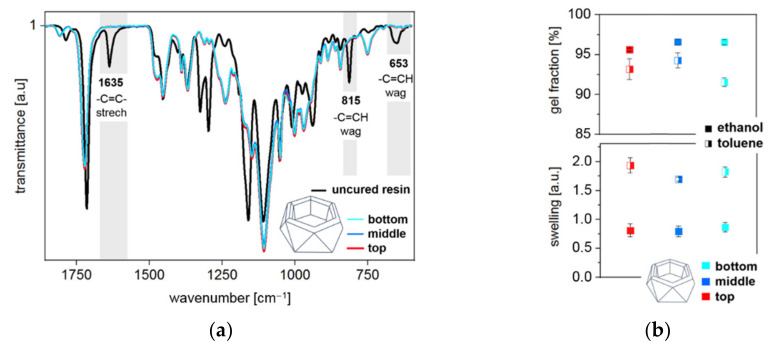
(**a**) Superimposed FT-IR spectra of the top (1–13 mm), middle (14–27 mm), and bottom (28–40 mm) regions are in exact congruence. Comparison with the uncured resin underlines high double bond conversion. (**b**) Swelling experiments were performed in toluene and ethanol. A slightly reduced gel-content is observed with toluene in the bottom region. Nevertheless, swelling, as well as gel-content, demonstrate uniformity over the sample depth.

Unlike the smooth areas that polymerized in contact with the silicone mold, the surface exposed to air remained slightly sticky due to oxygen inhibition. In view of a large number of existing solutions to this common problem, it is not addressed further within this study. To assess homogeneity, specimens were segmented into three parts (top 1–13 mm, middle 14–27 mm, and bottom 28–40 mm). Accumulated FTIR spectra of the individual sections demonstrate exact congruence and sufficiently high double bond conversion as indicated by the disappearance of peaks assignable to the double bond at 1635 (C=C stretch), 815 (C-H out of plane bending vibration) and 653 cm^–1^ (C-H out of plane bending vibration), respectively (Figure 6a). Apart from a slightly reduced gel-content in the bottom region in toluene, swelling, and gel-content are uniform over depth (Figure 6b).

### 3.3. Mechanical Characterization

**Figure 7:**
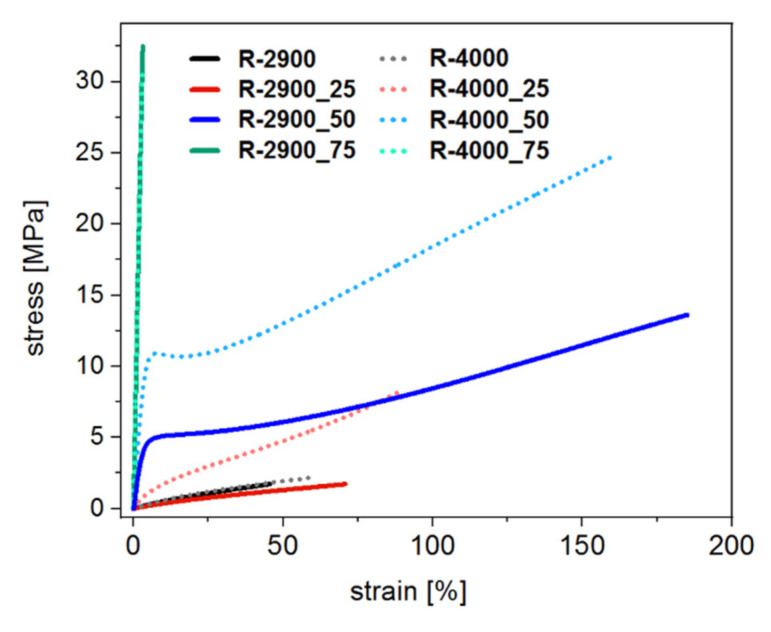
Exemplified stress vs. strain curves of polymers produced from the proposed FPP-resins. A substantial variation in mechanical properties is achieved through increasing copolymer content.

Light- sensitive resins have been optimized intensively within recent years, so that custom-made products, offering a broad range of characteristics are available nowadays. Many of these contain a significant proportion of absorbent fillers such as carbon black or ceramics since in 3D printing, increased absorption of the resin is often desired in keeping the viscosity low [8]. However, if a low optical density strategy is followed to maximize the depth of cure, absorbent fillers are avoided. Suitable methods for optimizing polymer properties include variation of the monomer content as well as toughening via living radical polymerization using chain transfer agents [36,37,38]. Polymers prepared from resins containing either PTHF_2900_-DM or PTHF_4000_-DM as crosslinkers and an increasing amount of IBOMA as copolymer are characterized to address, whether resins suitable for FPP, can be designed to meet various mechanical requirements. The polymers tested, exhibit mechanical properties, ranging from soft and rubbery (e.g., R-2900_25) to ductile (e.g., R-4000_50), as well as hard and brittle (e.g., R-2900_75) (Figure 7).

Polymers derived from pure crosslinkers (R-2900 and R-4000) show inferior mechanical properties. By adding IBOMA, the moduli increase to a maximum of 1123 MPa (R-4000_75) (Table 3). The addition of 75% IBOMA led to a hard and brittle behavior for R-2900 and R-4000. R-2900_75 exhibits similar modulus and tensile strength, and slightly higher elongation at break than R-4000_75 (Table 3).

Up to a concentration of 50% IBOMA, the elongation at break increases, with R-2900_50 reaching almost 200%. Comparing formulations containing 50% IBOMA, the difference in tensile strength and young’s modulus is exceptionally high. R-4000_50 is stronger on average, probably due to the proportion of MAA and MA.

## 4. Conclusions

This study underlines that resins suitable for frontal photopolymerization allow both, specific variation of mechanical properties and depth curing of several centimeters. Eight resins based on PTHF-DM copolymerized with an increasing amount of IBOMA were mechanically characterized and components produced in silicone molds. An increase in Young’s modulus from 5.3 to 1000 MPa was observed only by varying the copolymer content, with elongation at break reaching a maximum of approximately 200% with 50% IBOMA. The maximum tensile strength reached 32 MPa with 75% IBOMA. Photofrontally polymerized components with a mass of 60 g, and a depth of four centimeters demonstrate uniform double bond conversion. In addition, components with a mass of up to 267 g indicate that the possibilities of this technology are not yet exhausted.

## Figures and Tables

**Table 1:** Monomer composition of FPP-resins. Each resin contains 1.0 wt.% TPO-L.

FPP-Resin	PTHF_-_DM (wt.%)/(g/mol)	Isobornyl Methacrylate (wt.%)	Methacrylic Anhydride (wt.%)	Methacrylic Acid (wt.%)
R-2900	100.0/2900	-	-	-
R-2900_25	75.0/2900	25.0	-	-
R-2900_50	50.0/2900	5.0	-	-
R-2900_75	25.0/2900	75.0	-	-
R-4000	89.0/4000	-	7.0 ^1^	4.0 ^1^
R-4000_25	66.7/4000	25.0	5.3	3.0
R-4000_50	44.5/4000	50.0	3.5	2.0
R-4000_75	22.3/4000	75.0	1.7	1.0

^1^ The reaction mixture for PTHF_4000_-DM was used directly after modification so that excess MAA and MA, formed as a side product, function as copolymers. The indicated quantities were calculated, assuming a reaction yield of 100%.

**Table 2:** Exposure profile applied for specimen production.

Cycle	Intensity (%)	Runs	Exposure (J cm^−2^)	Total Exposure (J cm^−2^)
1	40	3	3.4	3.4
2	60	3	6.9	10.3
3	100	3	10.5	20.8

**Table 3:** Tensile properties of polymers produced with resins suitable for FPP.

FPP-Resin	Young’s Modulus (MPa)	Tensile Strength (MPa)	Elongation at Break (%)
R-2900	5.31 ± 0.14 ^1^	1.74 ± 0.12	45.3 ± 5.57 ^1^
R-2900_25	3.88 ± 0.13	1.76 ± 0.35	69.3 ± 15.0
R-2900_50	70.2 ± 10.6	13.7 ± 2.21	195 ± 39.5
R-2900_75	993 ± 61.1	32.0 ± 4.23	3.23 ± 0.41
R-4000	5.85 ± 0.23	2.11 ± 0.26	55.3 ± 10.8
R-4000_25	16.5 ± 1.57	8.12 ± 1.59	89.6 ± 11.8
R-4000_50	225 ± 8.37	24.0 ± 0.88	152 ± 9.29
R-4000_75	1129 ± 217	23.9 ± 6.73	2.18 ± 0.75

^1^ In a second series of tests, tensile specimens were fabricated with a translucent lid to avoid oxygen inhibition. Significantly higher Young’s moduli and lower elongation at break were observed; however, these specimens tended to have irregularities such as bubbles, which led to poor reproducibility. For better reproducibility, the specimens analyzed in this study were prepared without lids.

## Data Availability

The data presented in this study are available on request from corresponding author.

## Supplementary Materials

The following are available online at https://www.mdpi.com/1996-1944/14/4/743/s1, Figure S1: Photofrontally cured specimen with complex surface pattern and 3.0 cm depth, fabricated from 35 g of FPP resin R-2900_50. Figure S2: A total mass of 267 g resin polymerized via light induction only. The circular silicone mold of 13 cm diameter and 5.5 cm depth was extremely soft and flexible, so that the specimen is rather oval.
