# Supplementary material for: Resins for Frontal Photopolymerization: Combining Depth-Cure and Tunable Mechanical Properties
Source: Materials (Basel). 2021 Feb 5;14(4):743. doi: 10.3390/ma14040743 (PMC7914558; doi:10.3390/ma14040743)
Supplement: Supplementary file 1 [file materials-14-00743-s001.pdf]

materials

Supplementary Materials

Resins for Frontal Photopolymerization: Combining Depth-Cure and Tunable Mechanical Properties

Catharina Ebner, Julia Mitterer, Joamin Gonzalez-Gutierrez, Gisbert Rieß and Wolfgang Kern

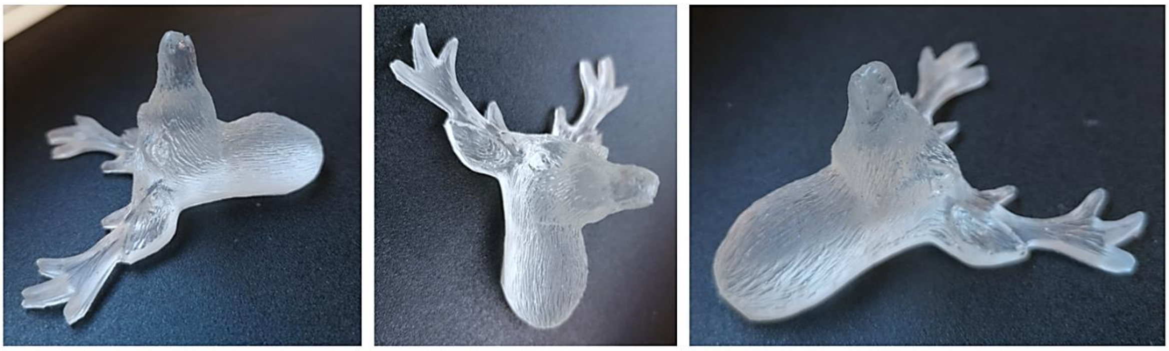

Figure S1. Photofrontally cured specimen with complex surface pattern and 3.0 cm depth, fabricated from 35 g of FPP resin R-2900_50.

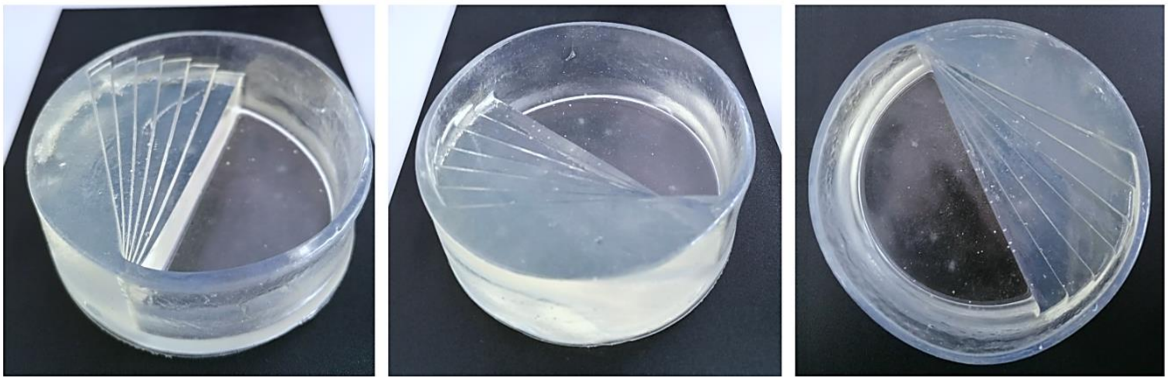

Figure S2. A total mass of 267 g resin polymerized via light induction only. The circular silicone mold of 13 cm diameter and 5.5 cm depth was extremely soft and flexible, so that the specimen is rather oval.
